# Antiviral Action of Synthetic Stigmasterol Derivatives on Herpes Simplex Virus Replication in Nervous Cells *In Vitro*

**DOI:** 10.1155/2014/947560

**Published:** 2014-07-24

**Authors:** Erina Petrera, Analía G. Níttolo, Laura E. Alché

**Affiliations:** Laboratorio de Virología, Departamento de Química Biológica, IQUIBICEN, Facultad de Ciencias Exactas y Naturales, Universidad de Buenos Aires, Pabellón 2, 4to. Piso, Intendente Güiraldes 2160, Ciudad Universitaria, C1428EGA Buenos Aires, Argentina

## Abstract

Polyfunctionalized stigmasterol derivatives, (*22S*,*23S*)-22,23-dihydroxystigmast-4-en-3-one (compound **1**) and (*22S*,*23S*)-3*β*-bromo-5*α*,22,23-trihydroxystigmastan-6-one (compound **2**), inhibit herpes simplex virus type 1 (HSV-1) replication and spreading in human epithelial cells derived from ocular tissues. Both compounds reduce the incidence and severity of lesions in a murine model of herpetic stromal keratitis when administered in different treatment modalities. Since encephalitis caused by HSV-1 is another immunopathology of viral origin, we evaluate here the antiviral effect of both compounds on HSV-1 infected nervous cell lines as well as their anti-inflammatory action. We found that both stigmasterol derivatives presented low cytotoxicity in the three nervous cell lines assayed. Regarding the antiviral activity, in all cases both compounds prevented HSV-1 multiplication when added after infection, as well as virus propagation. Additionally, both compounds were able to hinder interleukin-6 and Interferon-gamma secretion induced by HSV-1 infection in Neuro-2a cells. We conclude that compounds **1** and **2** have exerted a dual antiviral and anti-inflammatory effect in HSV-1 infected nervous cell lines, which makes them interesting molecules to be further studied.

## 1. Introduction

Herpesviruses are present throughout the animal kingdom having evolved over hundreds of millions of years with their hosts. To date, at least eight distinct human herpesviruses have been identified and, between them, herpes simplex virus (HSV) is the most ubiquitous. A characteristic of all herpesvirus infections, however, is their ability to establish lifelong infections characterized by periods of latency followed by reactivation.

For instance, the recurrent mucocutaneous infections exerted by HSV are painful and socially concerning; however, they are not the most serious manifestations of infection. Since HSV is so prevalent, complications of recurrence have a considerable medical burden on both immune competent and immune compromised persons. These complications include keratitis, hepatitis, pneumonia, esophagitis, and encephalitis [1].

The morbidity caused by these acute and recurrent infections produces much human suffering for which there remains an unmet need for control by effective immunization or antiviral therapy. To date, there has been little success with HSV vaccines. Antiviral drugs such as acyclovir (ACV) have been the mainstay of HSV therapy for three decades, with outstandingly few adverse effects. However, viral resistance has emerged as a potential problem which, together with the eradication of latency, has not been solved yet.

Previously, we have reported that some polyfunctionalized stigmasterol derivatives have* in vitro *antiviral activity with a broad spectrum of action. These synthetic compounds not only inhibit the multiplication of RNA viruses, such as Junin, Tacaribe, measles virus, and VSV, but also restrain HSV-1 multiplication [2–4]. Particularly, we have demonstrated that (*22S*,*23S*)-22,23-dihydroxystigmast-4-en-3-one (compound** 1**) and (*22S*,*23S*)-3*β*-bromo-5*α*,22,23-trihydroxystigmastan-6-one (compound** 2**) inhibit HSV-1 replication and spreading in human epithelial cells derived from ocular tissues [5, 6]. Both compounds reduce the incidence and severity of lesions in a murine model of HSV-1 induced ocular disease when administered in different treatment modalities [7]. This experimental model of ocular disease reproduces clinically and histologically the human herpetic stromal keratitis (HSK), an immunopathology of viral origin [8–10]. Furthermore, subsequent studies have revealed that the stigmasterol derivatives hinder the production of tumor necrosis factor alpha (TNF-*α*) in a LPS-stimulated murine macrophage cell line and modulate the secretion of Interleukin-6 (IL-6) and TNF-*α* in HSV-1 infected corneal and conjunctival cells, exerting an* in vitro* immunomodulatory effect [11, 12]. Hence, the improvement of HSK observed could also be ascribed to their immunomodulatory action [13].

One of the rare but devastating life-threatening diseases caused by HSV is adult and neonatal encephalitis (HSVE). Even though some therapies are available to treat patients with HSVE, as well as several antiviral drugs having been developed, outcomes still remain relatively poor [14]. Acyclovir treatment is effective in reducing mortality but is less effective in dismissing morbidity. Kamei et al. [15] have reported that the therapy combining acyclovir with corticosteroids appears to give a better outcome in adult patients with HSVE. One pharmacological mechanism related to corticosteroid in HSVE is the inhibition of proinflammatory cytokines such as IL-6 [16].

Considering the urgent need for different therapeutic options to combat acute and latent HSV infection in the brain, we decided to study the effect of both stigmasterol derivatives on HSV-1 replication in three nervous cell lines.

## 2. Materials and Methods

### 2.1. Cells, Viruses, and Treatment Solutions

Simian Vero cells were grown in Eagle's minimal essential medium supplemented with 5% inactivated fetal bovine serum (FBS) (MEM 5%) and 50 *μ*g/mL gentamycin. Human SH-SY5Y cell line was grown in Dulbecco's Modified Medium and Nutrient Mixture F-12 (DMEM/F12) 1 : 1 mixture, supplemented with 10% inactivated FBS (DMEM/F12 10%) and 50 *μ*g/mL gentamycin. Rat PC-12 cell line was grown in DMEM/F12 supplemented with 5% inactivated FBS, 10% inactivated horse serum, and 50 *μ*g/mL gentamycin. Murine Neuro-2a cell line was grown in DMEM/F12 10%, 50 *μ*g/mL gentamycin, and sodium pyruvate 5 *μ*g/mL.

HSV-1 Cgal+ (HSV-1 Cgal) KOS strain was propagated at low multiplicity on Vero cells. This system contains the* Escherichia coli lacZ *reporter gene encoding *β*-galactosidase under the control of a human cytomegalovirus promoter and was kindly provided by Dr. Alberto Epstein (Université Claude Bernard, Lyon, France).

The HSV-1 KOS wt strain was also propagated at low multiplicity.

Compounds** 1 **and** 2 **were dissolved in DMSO and diluted with culture medium for testing. The maximum concentration of DMSO tested (1%) exhibited no toxicity.

### 2.2. Cytotoxicity Assay

Cell viability in the presence of different concentration of the compound was determined using the MTT assay [17]. The absorbance of each well was measured on Eurogenetics MPR-A 4i microplate reader using a test wavelength of 570 nm and a reference wavelength of 630 nm. The CC_50_ was defined as the concentration of the compound that caused a 50% reduction in absorbance.

### 2.3. Antiviral Activity

Cells grown in 24-well plates were infected with HSV-1 KOS and, after virus adsorption, treated or not with the compounds. After 24 h of incubation at 37°C, supernatants were collected and titrated in Vero cells. Briefly, Vero cells grown in 24-well plates were infected with serial 10-fold dilutions of viral yields and incubated for 1 h at 37°C. Residual inocula were replaced by maintenance medium with 0.7% of methylcellulose. After 72 h of incubation at 37°C, cells were fixed with formaldehyde 10% and stained with Crystal Violet, and plaque forming units were counted.

### 2.4. Measurement of *β*-Gal Activity

Cells grown in coverslips inside 24-well plates were infected with HSV-1 Cgal. After adsorption, inocula were eliminated and cells were covered with MEM 1.5%. At 24 h after infection (p.i.), supernatants were harvested and stored at −70°C for titration, and cells were stained* in situ *for *β*-gal [5]. Coverslips were mounted, observed with an Olympus BX61 microscope, and photographed.

### 2.5. Indirect Immunofluorescence Assay

Cells monolayers grown on glass coverslips inside 24-well plates were fixed with methanol for 10 min at −20°C. After three washes with PBS, coverslips were incubated with diluted primary antibody for 30 min at 37°C and subjected to three additional washes with PBS. Afterwards, cells were incubated with secondary antibody for 30 min at 37°C. Finally, coverslips were rinsed, mounted, and photographed with an Olympus FB300 confocal microscope.

### 2.6. Cytokine Determination

Neuro-2a cells grown in 24-well plates were infected with HSV-1 at a multiplicity of infection (m.o.i.) of 1. After 24 h of incubation at 37°C cells supernatants were harvested and cytokines were quantified. Murine IL-6 and IFN-gamma were quantified by commercial ELISA sets (BD OptEIATM, Becton Dickinson, USA) according to manufacturer instructions.

### 2.7. Statistics

Data are presented as the means ± SD. Statistical significance was determined using Student *t*-test. A *P* < 0.05 was considered significant.

## 3. Results

### 3.1. HSV-1 Replication Kinetics in Nervous Cell Lines

Prior to the evaluation of the antiviral activity of the stigmasterol derivatives, we analyzed the kinetics of HSV-1 multiplication in three different cell lines.

Multistep kinetics were carried out in Neuro-2a, PC-12, and SH-SY5Y cells grown in 24-well plates, which were infected with HSV-1 strain KOS wt (m.o.i. = 1 PFU/cell). At 24, 48, and 72 h p.i., supernatants were harvested and virus yields were determined through a plaque assay in Vero cells.

**Figure 1:**
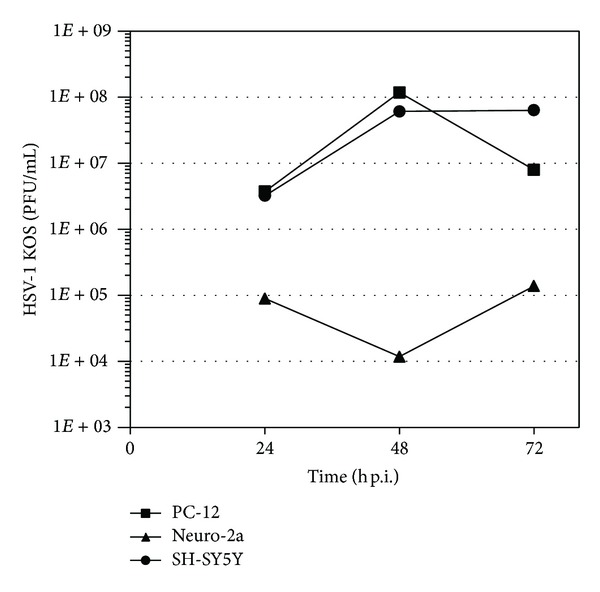
Effect of stigmasterol derivatives** 1** and** 2** on HSV-1 multiplication. PC-12 (square), Neuro-2a (triangle), and SH-SY5Y cells (circle) were infected with HSV-1 KOS (m.o.i.: 1) and incubated for 1 h at 37°C. After adsorption, cells were covered with DMEM/F12 2%. At 24, 48, and 72 h after infection supernatants were harvested and viral titres were determined by plaque assay. Data are mean values from two separate experiments ± SD.

As shown in Figure 1, HSV-1 replicated efficiently in these cultures. By 24 h p.i., viral titres were considerably lower in Neuro-2a cells, reaching a value of 10^5^ PFU/mL, whereas a value of 5 × 10^6^ PFU/mL was obtained for PC-12 and SH-SY5Y cells. By 48 h p.i., viral titres raised nearly two logs in both cell types, while a slight decrease in Neuro-2a cells was observed. In summary, PC-12 cells exhibited the highest susceptibility to HSV-1 infection and Neuro-2a and SH-SY5Y cells were also susceptible, reaching the maximum viral titres at 72 h (Figure 1). Interestingly, none of the three infected nervous cell lines exhibited cytopathic effect even after 72 h p.i. Taking into account previous findings and according to these results, we established 24 h p.i. and m.o.i. of 1 as parameters for HSV-1 infection in the three cell lines [5, 6].

### 3.2. Cytotoxicity of Compounds **1** and **2** in Nervous Cell Lines

Fifty percent cytotoxic concentration (CC_50_) for PC-12, Neuro-2a, and SH-SY5Y cells was determined. Compounds** 1** and** 2 **were added to confluent nongrowing cells in concentrations ranging from 1 to 144 *μ*M and from 1 to 129 *μ*M, respectively, and, after 24 h incubation at 37°C, a MTT colorimetric assay was performed.

**Figure 2:**
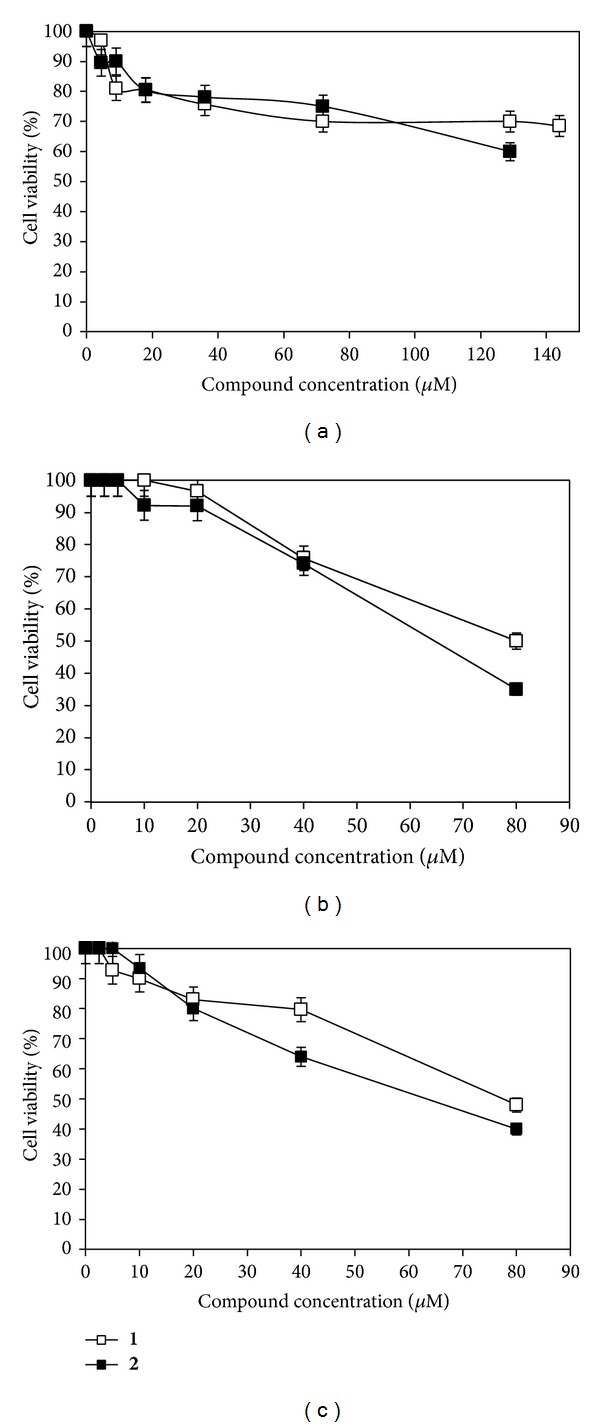
Cytotoxic effect of compounds** 1** and** 2** in three nervous cell lines. (a) PC-12, (b) Neuro-2a, and (c) SH-SY5Y cells monolayers were incubated in the presence of different concentrations of compounds** 1** and** 2**. After 24 h of incubation at 37°C cell viability was determined by the MTT assay. The CC_50_ was defined as the concentration of the compound that caused a 50% reduction in cell viability. Data are mean values from four separate experiments.

CC_50_ values of compounds** 1** and** 2** obtained in SH-SY5Y cells were 77.2 and 70 *μ*M, respectively (Figure 2(c)), similar to that previously reported for compound** 1** in IOBA-NHC cells (71.2 *μ*M) [6]. In the case of Neuro-2a (Figure 2(b)) and PC-12 (Figure 2(a)) cells treated with compound** 1**, CC_50_ values were even higher (80.4 and >144 *μ*M, resp.). On the other hand, CC_50_ values estimated for compound** 2** ranged from 65.2 in Neuro-2a cells to >129 *μ*M in PC-12 cells, and both were lower than that previously reported in IOBA-NHC cells (>277 *μ*M) (Figure 2) [5].

Hence, compound** 1** as well as compound** 2** displayed a low cytotoxicity for the three nervous cell lines assayed.

### 3.3. Anti-HSV Activity of Compounds **1** and **2** in Neuro-2a, PC-12, and SH-SY5Y Cells

Taking into account the broad spectrum of antiviral activity already reported for compounds** 1** and** 2 **[2–6, 18, 19] we decided to study their anti-HSV-1 effect in nervous cell lines, which were infected with two different strains of HSV-1, KOS wt strain, and KOS Cgal strain.

To assay the antiviral activity of compounds** 1** and** 2**, we chose concentrations of 10 *μ*M and 5 *μ*M, respectively, which were, at least, seven times lower than the corresponding CC_50_ values found (Figure 2). Neuro-2a, PC-12, and SH-SY5Y cells grown in 24-well plates were infected with HSV-1 KOS wt strain at m.o.i. of 1. After 24 h of incubation at 37°C, supernatants were collected and titrated in Vero cells.

**Figure 3:**
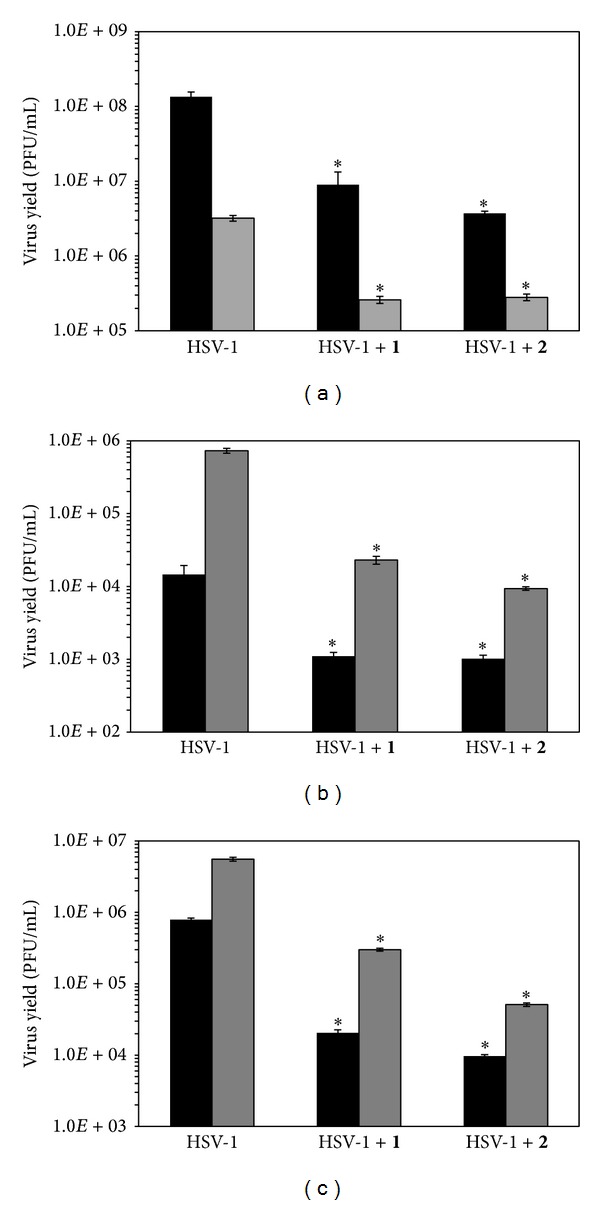
Effect of compounds** 1** and** 2 **on HSV-1 virus yield reduction. (a) PC-12, (b) Neuro-2a, and (c) SH-SY5Y cells were infected with HSV-1 KOS (black bars) and HSV-1 Cgal (grey bars) and incubated for 1 h at 37°C. Then, cells were covered with DMEM/F12 2% or media containing 10 *μ*M of compound** 1 **and 5 *μ*M of compound** 2**. After 24 h supernatants were harvested and viral titres were determined by plaque assay. Bars show average of two replicates ± SD.  *P < 0.05, with respect to untreated infected cells.

HSV-1 Cgal strain multiplied more efficiently than KOS wt strain in Neuro-2a and SH-SY5Y cells, whereas viral titre was significantly lower in PC-12 cells (Figures 3(a), 3(b), and 3(c)).

Both compounds restrained HSV-1 KOS wt multiplication since viral titres decreased in around 1-2 logs with respect to viral titres obtained from untreated infected cells. Compound** 2** was more efficient than compound** 1** to restrain HSV-1 yield in the three nervous cell lines tested, reaching an inhibition of nearly 2 logs in SH-SY5Y cells (Figures 3(a), 3(b), and 3(c)). Thus, both compounds prevented HSV-1 KOS wt multiplication in the three nervous cell lines used when added after infection.

We also found that recombinant HSV-1 Cgal was able to multiply in PC-12, Neuro-2a, and SH-SY5Y cells and, regarding the antiviral activity, both compounds hindered HSV-1 Cgal replication in all cases (Figures 3(a), 3(b), and 3(c)). Compounds** 1 **and** 2 **caused 1-2 log reduction in viral yields regardless of the virus strain used.

**Figure 4:**
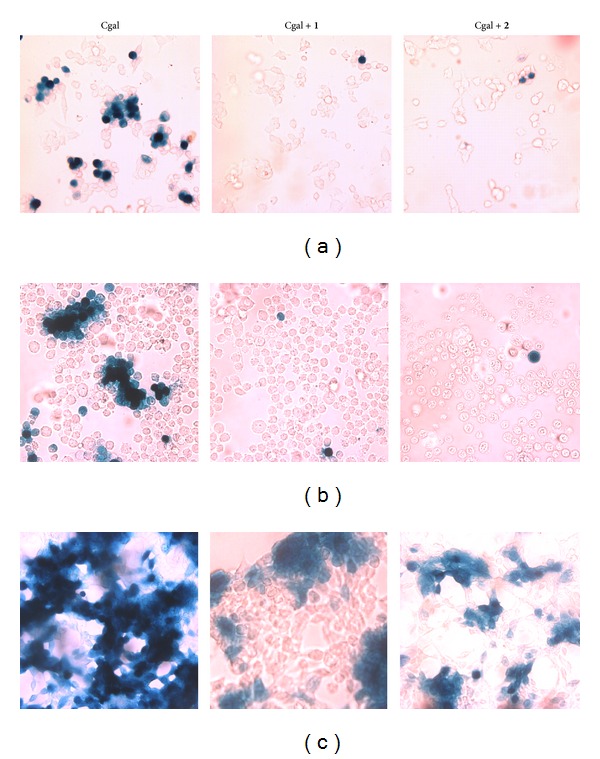
Effect of compounds** 1** and** 2 **on HSV-1 propagation. (a) PC-12, (b) Neuro-2a, and (c) SH-SY5Y cells monolayers grown in coverslips inside 24-well plates were infected with HSV-1 Cgal and incubated for 1 h at 37°C. Then, cells were covered with DMEM/F12 2% or media containing 10 *μ*M of compound** 1 **and 5 *μ*M of compound** 2**. After incubation for 24 h, cells were stained* in situ* for *β*-gal. Cgal: HSV-1 Cgal infected-untreated cells; Cgal+1: HSV-1 Cgal infected** 1**-treated cells; Cgal+2: HSV-1 Cgal infected** 2**-treated cells. Magnification: 40x.

We have previously reported that compounds** 1 **and** 2** inhibit HSV-1 propagation in IOBA-NHC cells [5, 6]. To investigate the effect of both compounds on viral propagation, PC-12, Neuro-2a, and SH-SY5Y cells grown in coverslips inside 24-well plates were infected with HSV-1 Cgal (m.o.i. = 1) and, then, treated with control media, 10 *μ*M of** 1 **and 5 *μ*M of** 2**. After 24 h incubation at 37°C, cells were stained* in situ *for *β*-gal. In the absence of compounds, stained cells clustered in characteristic HSV-1 foci were observed (Figure 4), whereas all infected cells treated with either** 1 **or** 2 **exhibited only scattered stained cells (Figure 4). Therefore, both compounds would prevent HSV-1 Cgal spreading in all nervous cell lines assayed when added after infection.

**Figure 5:**
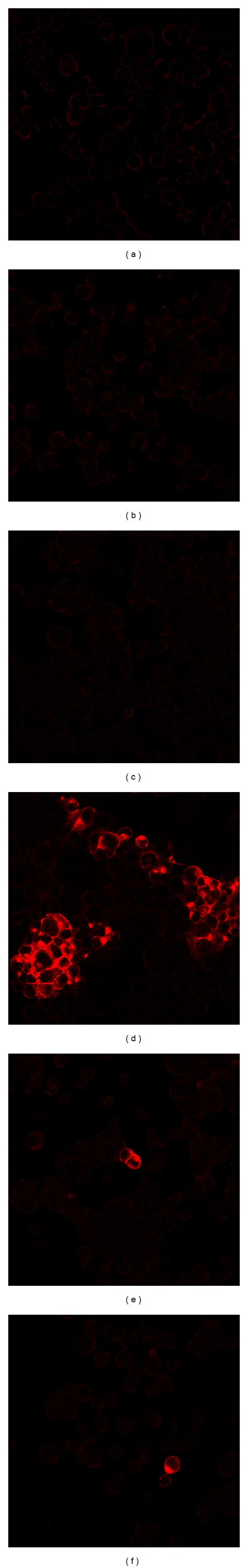
Effect of compounds** 1** and** 2** on HSV-1 KOS multiplication in three nervous cell lines. Neuro-2a cells grown in coverslips inside 24-well plates were infected with HSV-1 KOS wt and incubated for 1 h at 37°C. Then, cells were covered with DMEM/F12 2% or media containing 10 *μ*M of compound** 1 **and 5 *μ*M of compound** 2**. After 24 h p.i., cells were fixed with methanol and HSV-1 gD localization was done by IFI staining. (a) Control cells; (b) mock-infected cells treated with compound** 1**, (c) mock-infected cells treated with compound** 2**, (d) HSV-1 KOS infected cells, (e) HSV-1 KOS infected cells treated with compound** 1**, and (f) HSV-1 KOS infected cells treated with compound** 2**. Magnification: 40x.

To corroborate these results, an indirect immunofluorescence was performed. For that purpose, Neuro-2a cells monolayers grown in coverslips inside 24-well plates were infected with HSV-1 KOS (m.o.i. = 1) and then treated with control media, 10 *μ*M of** 1** and 5 *μ*M of** 2**. After 24 h of incubation at 37°C, supernatants were removed and cells were washed and fixed as described in Materials and Methods. Cells were incubated with mouse monoclonal antibodies anti-gD of HSV-1 and revealed with anti-mouse FluoroLinkTM CyTM3 antibodies. The inhibition of viral spread caused by both compounds shown in Figure 5 confirmed the restriction of HSV-1 propagation previously observed (Figure 4).

### 3.4. Anti-Inflammatory Effect of Compounds **1** and **2** in HSV-1-Infected Neuro-2a Cells

We have previously reported that compound** 1** stimulates anti-inflammatory molecules and inhibits proinflammatory factors in macrophages activated with a nonviral stimulus, suggesting an immunosuppressive action over inflammatory cells [13]. On the other hand, compound** 2** increases IL-6 levels in human epithelial cells infected with HSV-1 [12]. Hence, we determined cytokine levels in HSV-1 KOS-infected Neuro-2a cells after treatment with both compounds, by ELISA. For that purpose, we used those supernatants in which the antiviral effect of compounds** 1** and** 2** was found (Figure 3(b)). IL-6 and IFN-*γ* secretion reached 659.6 ± 14.8 pg/mL and 154.7 ± 7.2 pg/mL in HSV-1 infected control cells, respectively. IL-6 levels were reduced in 46.2% (355.4 ± 10.9 pg/mL, *P* < 0.001) and 41.1% (388.2 ± 11.4 pg/mL, *P* < 0.001) when infected cells were treated with compounds** 1** and** 2**, respectively. IFN-*γ* levels dropped to 72.3 ± 4.9 pg/mL (*P* < 0.006) (53.3%) and to 53 ± 4.2 pg/mL (*P* < 0.001) (65.7%) with respect to untreated infected cells after treatment with** 1** and** 2**. Thus, compounds** 1 **and** 2 **might display an anti-inflammatory activity in HSV-1-infected Neuro-2a cells.

## 4. Discussion

Many viruses of public health significance may cause disease by triggering an immunopathology in which the damage is produced by the host inflammatory response elicited by the virus [20].

In the general population, HSV is highly prevalent (more than 70% after age of 50). This virus persists latently in the peripheral nervous system and periodically reactivates with production of active virus. The pathogenic mechanisms of HSV-1 at the central nervous system (CNS) are not well known. The virus enters the brain and infects neurons, where recurrent reactivations of HSV in CNS of adult people could happen [21, 22]. On the other hand, HSVE is a rare but very severe acute infection of the CNS. Brain inflammation due to infection is associated with the activation of the local innate immune system. This could be a crucial mechanism leading to the neuronal damage, as it was also described in the case of HSK, where a chronic inflammatory reaction in response to viral reactivation in the eye may lead to vision impairment and even blindness [8–10, 23].

Although intravenous ACV blocks viral replication and significantly reduces the mortality associated with HSVE, many infected patients still suffer from severe neurological sequelae [24]. In turn, though progression of human HSK is not prevented by ACV, treatment of HSK also includes ACV to mitigate viral reactivation due to the immunosuppression caused by corticosteroids, which are supplied to alleviate the symptoms of disease [25].

Corticosteroids may be also beneficial in HSVE for decreasing the activation of several inflammatory pathways even though their adverse side effects are well known [26]. It was reported that combined therapy using both ACV and corticosteroids can achieve a better outcome in adult patients with HSVE as well as reducing the progression of inflammation during HSK [10, 15]. The healing effect of compounds** 1** and** 2** on the evolution of HSV-1-induced ocular disease in mice reveals their dual effect, antiviral and anti-inflammatory [5, 6].

The present study clearly demonstrates that stigmasterol derivatives** 1 **and** 2** have inhibited HSV-1 multiplication and propagation in nervous cell lines of different origin (Figures 3, 4, and 5), which is consistent with the already reported antiherpetic activity observed in other cell lines [2, 5, 6, 18, 19].

On the other hand, the anti-inflammatory effect of the compounds varied depending upon cell substrate. Compound** 1** does not reduce TNF-*α* and IL-6 levels in HSV-1-infected epithelial cells, whereas compound** 2 **inhibits TNF-*α* in IOBA-NHC cells and increases IL-6 levels in HSV-1-infected IOBA-NHC and HCLE cells [12] (Michelini, personal communication). Furthermore, many overexpressed and repressed genes associated with innate immune responses and inflammatory processes have been detected in HSV-1-infected epithelial cells and activated macrophages treated with compound** 1**, by means of RNA microarrays [13].

It is well-known that IL-6 has mainly proinflammatory activity and plays an important role in inflammatory processes in the CNS [16, 27, 28]. Thus, an increased expression of IL-6 mRNA in brain tissue of mice infected with HSV was shown during the first three weeks after infection [27]. In fact, a significant increase of IL-6 concentration in CSF of patients with HSVE was also observed [16, 28, 29].

IFN-*γ* is also involved in the development of CNS pathologies since it enhances the function of microglia by increasing the production of TNF-*α*, NO, and free radicals, which play critical roles in the progress of inflammatory demyelination and neuronal degeneration [30].

Herein, our findings clearly indicate that compounds** 1** and** 2** have been able to inhibit the secretion of IL-6 and IFN-*γ* induced by HSV-1 KOS wt infection of Neuro-2a cells. Although the anti-inflammatory effect of compounds** 1** and** 2** observed may be ascribed to their antiherpetic activity, we have reported that both compounds are able to reduce IL-6 secretion in macrophages induced with a nonviral stimulus, in a dose-dependent manner [12].

In an effort to reduce neuronal damage secondary to host inflammatory response, cytokine modulation is an avenue that has been proposed for the treatment of HSVE. Thus, the effect of glucocorticoids was evaluated in a mouse model of HSVE and revealed that modulating the innate immune response in a timely manner could prevent neurological damage and mortality [31].

Although compounds** 1** and** 2** showed a structural similitude with the commercial anti-inflammatory glucocorticoid dexamethasone, none of them was able to bind glucocorticoid receptors (GR) or induce a glucocorticoid activity via GR activation (Michelini, personal communication). Thus, they would be activating a different signaling pathway from that of glucocorticoids, exerting their anti-inflammatory activity through a different mechanism of action. This finding constitutes an advantage in the use of these compounds over glucocorticoids because they would not exert the known undesirable side effects of the latter.

## 5. Conclusions

It is the first time that compounds** 1** and** 2** prove to have a significant antiviral and anti-inflammatory effect in HSV-1 infected nervous cell lines from different sources. Taking into account that compound** 1** has been recently patented as a compound showing anti-inflammatory and antiviral activities to treat HSK, it deserves to be further studied as a potential tool to limit CNS diseases caused by HSV.
